# Theoretical Study of the BaTiO_3_ Powder’s Volume Ratio’s Influence on the Output of Composite Piezoelectric Nanogenerator

**DOI:** 10.3390/nano7060143

**Published:** 2017-06-09

**Authors:** Xi Zhou, Qi Xu, Suo Bai, Yong Qin, Weisheng Liu

**Affiliations:** 1 Key Laboratory of Nonferrous Metals Chemistry and Resources Utilization of Gansu Province, State Key Laboratory of Applied Organic Chemistry, College of Chemistry and Chemical Engineering, Lanzhou University, Lanzhou 730000, China; zhoux13@lzu.edu.cn; 2 Institute of Nanoscience and Nanotechnology, School of Physical Science and Technology, Lanzhou University, Lanzhou 730000, China; xuq10@lzu.edu.cn (Q.X.); baisuo@lzu.edu.cn (S.B.); 3 School of Advanced Materials and Nanotechnology, Xidian University, Xi'an 710071, China

**Keywords:** piezoelectric nanogenerator, composite material, optimal volume ratio, performance optimization

## Abstract

The combination of the piezoelectric materials and polymer is an effective way to make the piezoelectric nanogenerator (PENG) possess both the polymer’s good flexibility and ferroelectric material’s high piezoelectric coefficient. The volume ratio of ferroelectric material in the composite is an important factor that determines the PENG’s output performance. In this paper, the BaTiO_3_/polydimethylsiloxane (PDMS) composite PENG was demonstrated as having an optimal volume ratio (46%) at which the PENG can output its highest voltage, and this phenomenon can be ascribed to the trade-off between the composite PENG’s top electrode charge and its capacitance. These results are of practical importance for the composite PENG’s performance optimization.

## 1. Introduction

With the development of nanotechnology and micro-electromechanical systems (MEMS), the implantable medical devices, personal electronics, and distributed networks have been widely explored to facilitate people’s daily life. Despite the convenience brought by these technologies, some potential problems begin to emerge because of the limited capacity of traditional batteries used in the above devices. It is especially inconvenient to replace depleted batteries for implantable medical devices because it is a miserable experience for patients and a high risk surgical procedure for surgeons. Even worse, among the numerous nodes in distributed sensor networks, it is almost impossible to find the nodes running out of power. To solve the above-mentioned problems, piezoelectric nanogenerator [1], which can harvest abundant in vivo, in vitro body motion energy and environmental mechanical energy, is developed to provide power supply for the implantable medical devices [2,3,4,5,6,7], personal electronics [8,9,10,11,12,13,14,15], and distributed sensor networks. As a power supply, the electric output and endurance are two key issues for piezoelectric nanogenerator’s (PENG’s) practical applications. Composite PENG, based on inorganic piezoelectric material and organic polymer, is a promising candidate self-powered generators with both good flexibility and high piezoelectric coefficient.

For the preparation of composite PENG, the key issues to be solved are how to disperse the ferroelectric materials uniformly in the polymer matrix and to find out at which ratio of the ferroelectric material, the PENG can output the maximum electricity. Due to the polymer matrix’s sticky and hydrophobic properties, it is hard to obtain a well-distributed composite by dispersing the ferroelectric material directly into the polymer matrix. In order to solve these problems, multiwalled carbon nanotubes [16,17], Cu nanorods [18], ZnS nanorods [19], virus templates [20], and bacterial cellulose [21] fillers have been used to facilitate the dispersion of the ferroelectric material, because the ferroelectric material can form a complex mixture with filler-based-entangled network structure. Some of the research results showed the existence of an optimal ratio in the ferroelectric material’s ratio in the composite PENG [18,21,22]. Therefore, systematic theoretical research is needed to study this phenomenon for the development of composite PENG.

In this paper, we studied the output voltage of the BaTiO_3_/PDMS composite PENG with different volume ratios of evenly distributed BaTiO_3_ cubes, and found the existence of an optimal volume ratio (46%) of the BaTiO_3_ cubes at which the PENG can output the highest voltage, resulting from the trade-off between the surface charge and capacitance of the composite PENG.

## 2. Methods and Structure

To obtain the composite PENG’s open circuit voltage, we need to solve the following governing equations for piezoelectric materials:

*σ*_p_ = *c*_pq_ε_q_ − *e*_kp_*E*_k_, (1)

*D_i_* = *e*_iq_*ε*_q_ + *κ_i_*_k_*E*_k_, (2)

where *σ*_p_ is the stress tensor, and *ε*_q_ is the strain tensor. To keep the tensor equation compact, the Voigt notion was used to reduce the 3 × 3 symmetric stress tensor *σ*_mn_ and strain tensor *ε*_mn_ where *m*, *n* ∈ (*x*, *y*, *z*), to 6-dimensional vectors *σ*_q_ and *ε*_p_ where *q*, *p* ∈ (*xx*, *yy*, *zz*, *yz*, *zx*, *xy*), *c*_pq_ is the linear elastic constant, *e*_kp_ is the linear piezoelectric coefficient, *κ_i_*_k_ is the dielectric constant, *E*_k_ is the electric field, and *D_i_* is the electric displacement. In this study, these equations are solved by the COMSOL software package (5.1, COMSOL Co., Ltd., Shanghai, China).

**Figure 1:**
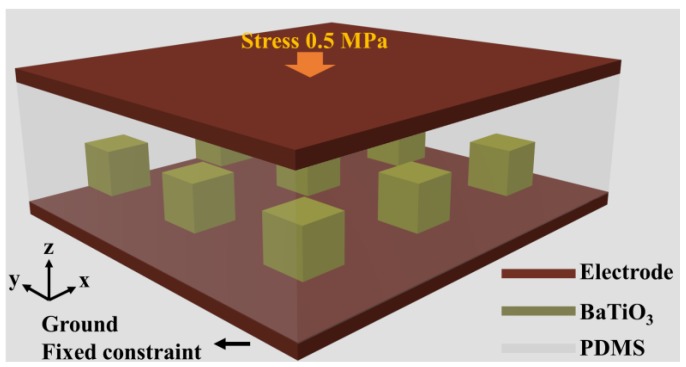
Schematic illustration of the BaTiO_3_/polydimethylsiloxane (PDMS) composite piezoelectric nanogenerator (PENG).

The structure of the simulated BaTiO_3_/PDMS composite PENG is schematically shown in Figure 1, where the BaTiO_3_/PDMS composite is sandwiched between the top and bottom electrodes. The size of the PDMS matrix is 460 × 460 × 40 μm, and the size of the individual BaTiO_3_ cube poling along the *z* axis is 30 × 30 × 30 μm, and the cubes are uniformly distributed in the matrix. In practice, it is inevitable for the BaTiO_3_ particles/polymer based PENG to have polymer between the electrode and BaTiO_3_. As shown in Figure 1, PDMS layer between the electrode and the BaTiO_3_ cubes in 5 μm thickness was used to mimic this situation. In practical calculations, the electrodes are not added, the bottom of the composite was ground and fixed, the charge on the side walls and top is set to be zero, a stress of 0.5 MPa is exerted on the top surface. BaTiO_3_/PDMS composite containing, 9, 16, 25, 36, 49, 64, 81, 100, 121, 144, 169, 196, and 225 BaTiO_3_ cubes are respectively calculated. The corresponding volume ratios are 2.9%, 5.1%, 8.0%, 11.5%, 15.6%, 20.4%, 25.8%, 31.9%, 38.6%, 45.9%, 53.9%, 62.5%, and 71.8%, respectively. After the calculation, the average electric potential of the top surface is the PENG’s open circuit voltage.

## 3. Results and Discussion

**Figure 2:**
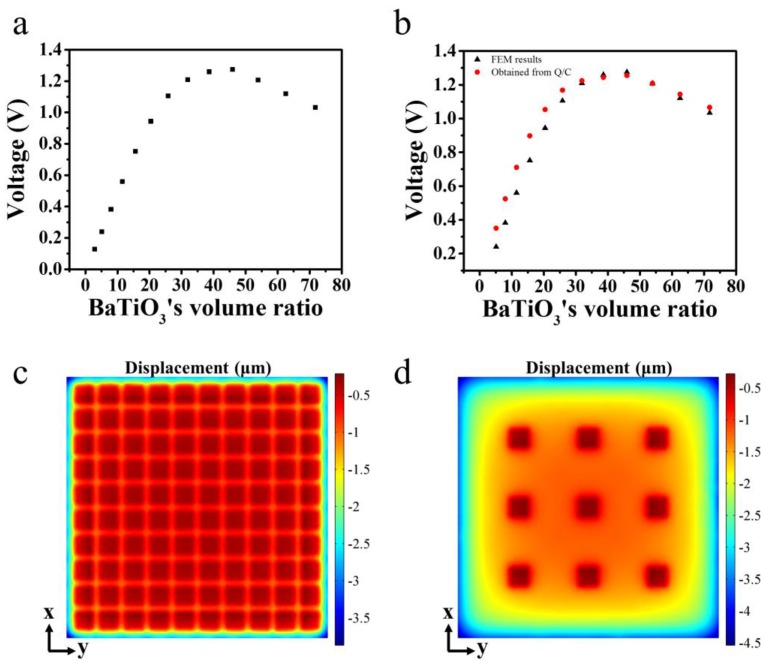
(**a**) The open circuit voltage of the composite PENG containing different volume ratios of BaTiO_3_ cubes; (**b**) The comparison of the voltage obtained *Q*/*C* and that directly calculated from the coupled piezoelectric governing equation. The distribution of stress on the BaTiO_3_ cubes’ top surface with volume ratio of 31.9% (**c**) and 2.9% (**d**).

As shown in Figure 2a, the open circuit voltage of BaTiO_3_/PDMS composites with different volume ratios of BaTiO_3_ cubes are calculated. At the volume ratio of 45.9%, the composite PENG reached its max voltage of 1.275 V. To interpret this phenomenon, the composite PENG is approximated as a capacitor whose charge is generated by piezoelectric effects. Below this approximation, the voltage of the PENG can be calculated.

*V* = *Q*/*C* (3)

where, *Q* is the electric charge and *C* is the composite PENG’s capacitance. The accuracy of this approximation is examined by comparing the voltage calculated by *Q*/*C* with that obtained directly by solving the coupled piezoelectric governing equation through finite element method (FEM). As shown in Figure 2b, we can see this approximation has a rather high accuracy when the BaTiO_3_ cubes’ ratio is larger than 31.9%. Below this volume ratio, the voltage obtained by *Q*/*C* is higher than that obtained by solving the coupled piezoelectric governing equation. This discrepancy is due to the fact that the external forces on the deformation of PENG are not considered. When the volume ratio of BaTiO_3_ cubes is 31.9%, the composite is stiff and the PENG’s deformation under external force is small, as shown in Figure 2c. In contrast, when the volume ratio of the BaTiO_3_ cubes in the composite is low, under the external force, the deformation of the PDMS filling between the BaTiO_3_ cubes is large as shown in Figure 2d. The capacitance of the plate capacitor is

*C* = *ε*_0_*κS*/*d* (4)

where, *ε*_0_ is the vacuum permittivity, and *κ* is the composite’s permittivity. The calculated capacitance of the PENG with low BaTiO_3_ cubes is smaller than the actual value without consideration of the deformation of the PENG under external force. Therefore, the voltage calculated on the basis of this capacitance is larger than the actual value. With the increase of BaTiO_3_ cubes’ volume ratio, the composite becomes stiffer and stiffer, so the PENG’s deformation can be neglected and the voltages calculated by the above two methods is consistent, as shown in Figure 2b. As the optimal volume ratio locates at 45.9%, it is sufficient to interpret this phenomenon by comparing the volume ratio ranging from 31.9% to 71.8%. In this volume ratio range, the Equation (3) has a rather good accuracy in describing the PENG’s voltage.

**Figure 3:**
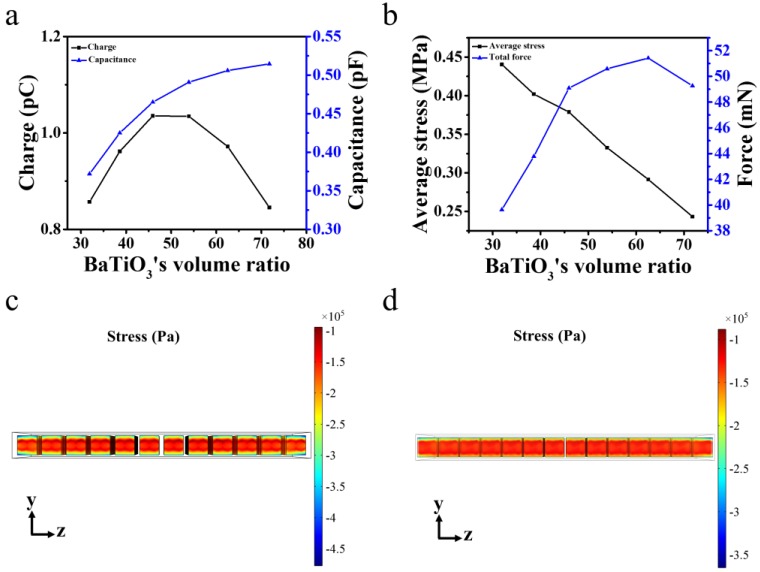
(**a**) The PENG’s top surface charge and capacitance with different BaTiO_3_ ratios. (**b**) The averaged stress and total force on the top surface of the embedded BaTiO_3_ cubes. The distribution of stress in the BaTiO_3_ at 45.9% (**c**) and 62.5% (**d**).

Results of the surface charge and the capacitance of the PENG are shown in Figure 3a. With the increase of BaTiO_3_ cube’s ratio, the value of PENG’s capacitance increases gradually and toward a saturated value, because the permittivity of BaTiO_3_ is much higher than that of the PDMS. In contrast, the surface charge first increases with the BaTiO_3_’s ratio and then decreases when the BaTiO_3_’s ratio is further increased, because the surface charge of the composite PENG is induced by the charge generated by the BaTiO_3_ cubes under external force and the electric charge in the ferroelectric materials composed of two parts [23]. One is the surface charge caused by the discontinuous polarization across the ferroelectric material’s surface and this value is proportional to the surface stress exerted on the ferroelectric material. The other one is the body charge caused by the gradient of the polarization, and this value is proportional to the gradient of the stress field in the ferroelectric material. The calculated average pressure and total force on the BaTiO_3_ cube’s top surface are shown in Figure 3a. When the BaTiO_3_’s ratio is increased, the average stress applied on the BaTiO_3_ cubes decreases, because the BaTiO_3_ cube in the polymer matrix has the property to concentrate the stress on it. As the cubes increase, the stress is more dispersed. On the one hand, the total force applied on the BaTiO_3_ cubes reaches its max at the volume ratio of 62.5%. On the other hand, as the BaTiO_3_ cubes’ ratio increases, the stress gradient in BaTiO_3_ cubes decreases. So the charge of the top has a maximum within this volume ratio range. With the trade-off of the capacitance and top electrode charge, the composite PENG reached its maximum output, when the BaTiO_3_ ratio is 45.9%.

**Figure 4:**
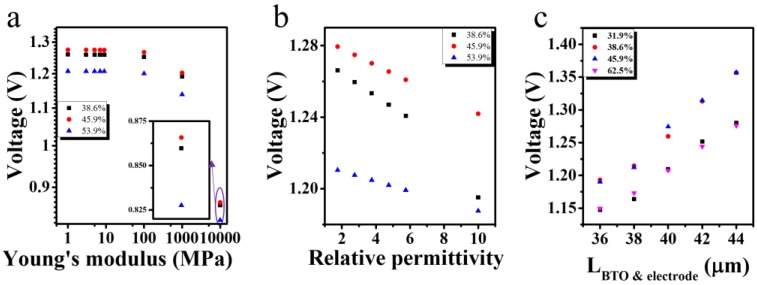
The open circuit voltage of the PENG at different polymer Young’s modulus (**a**), permittivity (**b**), and the distance between electrode and BaTiO_3_ cubes (**c**).

In the experiment, the polymer’s Young’s modulus and permittivity are not unique as they are often influenced by experimental conditions such as the amount and temperature of curing agent. In order to examine whether the optimal volume ratio changes with these uncertain factors, the open circuit voltage of PENG with BaTiO_3_ cubes volume ratio around the optimal value is studied. The results are shown in Figure 4a,b. When the polymer’s permittivity was increased, the voltage decreased notably, which is ascribed to the PENG’s increased capacitance. Although the voltage of the PENG is altered when the polymer’s Young’s modulus or permittivity are changed, the PENG with BaTiO_3_ volume ratio of 45.9% still has the max voltage. According to the experimental results [21] published recently, a paper-based PENG composed of BaTiO_3_ nanoparticles and bacterial cellulose got its maximum voltage when the mass ratio of uniformly distributed BaTiO_3_ nanoparticles reached 80%. The Young’s modulus, relative permittivity, and density of the bacterial cellulose are 10 GPa, 7.8, 1.2 g/cm^3^ respectively [24,25]. The PDMS’s Young’s modulus and relative permittivity are tuned to 10 GPa and 7.8, the optimal volume ratio of BaTiO_3_ is still 45.9% which can be seen in Figure 4a,b. In consideration of the density of BaTiO_3_ and bacterial cellulose at 6.02 g/cm^3^ and 1.2 g/cm^3^, the optimal mass ratio is 81%. Therefore, our simulation results are in consistent with those experimental results. The distance between the electrode and the BaTiO_3_ cubes was studied. As shown in Figure 4c, the PENG’s voltage increases with the increase of this distance, which may be ascribed to more stress delivered from the PDMS to the BaTiO_3_ cubes.

**Figure 5:**
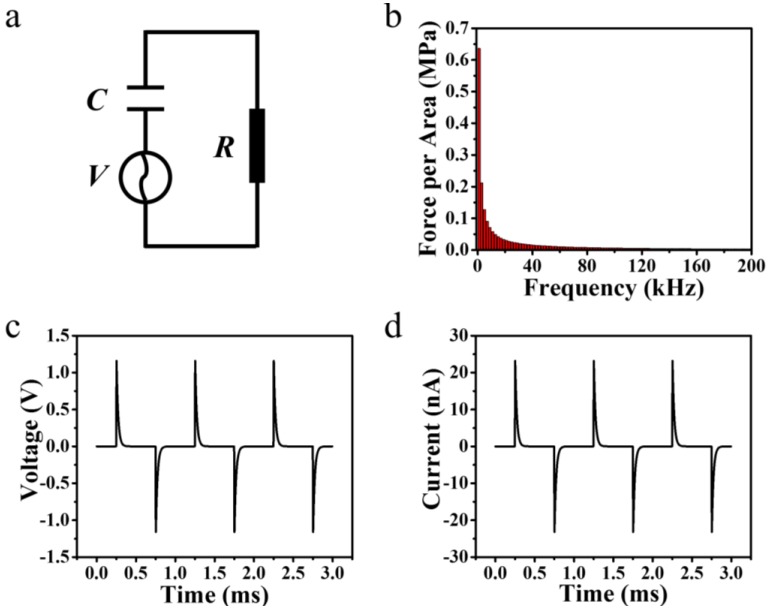
(**a**) The equivalent circuit of the composite PENG. (**b**) The spectral analysis of the square wave force. The output voltage (**c**) and current (**d**) at an external load with a resistance of 50 MΩ.

To convert the mechanical energy into electricity, a dynamic force must be exerted on the PENG. So the output of the PENG under the dynamic force was studied. A periodic square wave form force with an amplitude of 0.5 MPa was exerted on the PENG. According to the previous studies, a simple RC circuit with the PENG as the voltage source as shown in Figure 5a can be used to describe the PENG’s dynamic electric characters [26], the output of the voltage source can be approximated with a quite high accuracy as [27]

*V*(*t*) = *d*_33_*f*(*t*)/*C* (5)

where, *d*_33_ is the piezoelectric constant of the PENG, and *f*(*t*) is the dynamic force exerted on the PENG. When the PENG is driven by a harmonic force *f*(*t*)*e^iωt^*, where *ω* is the angular frequency of the force, the voltage drop across the external load has an analytic expression,

(6) $$V_{\omega }(t)=\frac{d_{33}f_{\omega }(t)R}{RC+\frac{1}{i\omega }}e^{i\omega t}$$

Thus, it is convenient to do a spectral analysis of the periodic square wave form force to obtain the voltage drop across the external load. After a spectral analysis of the square wave form force as shown in Figure 5b, the PENG’s voltage and current output at an external resistance of 50 MΩ were calculated as shown in Figure 5c,d. Under this force, the current and voltage have respective peak values of 1.16 V and 23.2 nA, and the peak power of this PENG is 26.9 nW.

## 4. Conclusions

In this paper, we studied the output voltage of the BaTiO_3_/PDMS composite PENG with different volume ratios of evenly distributed BaTiO_3_ cubes, and find that an optimal volume ratio (46%) of the BaTiO_3_ cubes exists at which the PENG can output the highest voltage. The optimal ratio is a result of the trade-off between the surface charge and capacitance of the composite PENG. This optimal volume ratio is stable even if the Young’s modulus and permittivity of the polymer matrix are changed. Finally, after a 0.5 MPa, 1000 Hz square wave force was exerted, the PENG at the optimal volume ratio can output a current of 23.2 nA, voltage of 1.16 V on the external load with resistance of 50 MΩ.
